# Supplementary material for: Post-translational modification-dependent oligomerization switch in regulation of global transcription and DNA damage repair during genotoxic stress
Source: Nat Commun. 2024 May 15;15:4128. doi: 10.1038/s41467-024-48530-8 (PMC11096357; doi:10.1038/s41467-024-48530-8)
Supplement: Supplementary file 1 — Supplementary Information [file 41467_2024_48530_MOESM1_ESM.pdf]

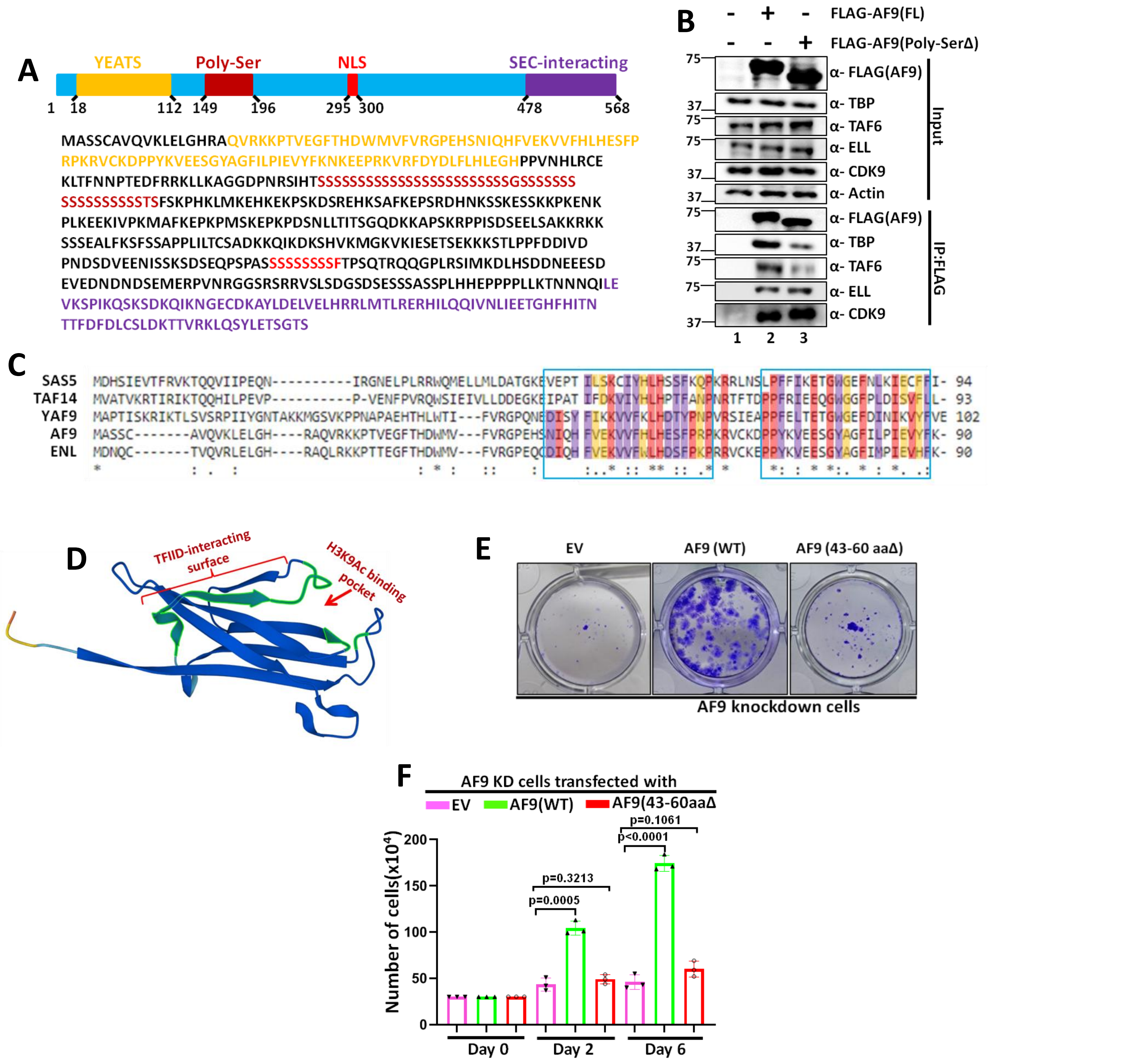

Supplementary Figure 1: Both Poly-Ser and YEATS domain of AF9 are important for its functional regulation within 293T cells.

A. Cartoon diagram along with corresponding amino acid sequences showing functionally important domains of human AF9.

B. Immunoblotting analysis showing interaction of ectopically-expressed full-length AF9 and Poly-SerΔAF9 with indicated TFIID and SEC components. 293T cells were transfected with plasmids expressing indicated proteins and interaction with TFIID and SEC components were analyzed by immunoprecipitation with M2 FLAG-agarose beads followed by western blotting analysis using factor-specific antibodies as indicated (n=3 replicates).

C. Alignment of YEATS domain of indicated proteins by using Clustal Omega tool showing sequence conservation between 43-60 amino acids across multiple YEATS domain-containing proteins.

D. Functionally distinct domain surfaces present within the YEATS domain for its interaction with TFIID as well as histone H3K9 acetylated peptide as described in the PDB entry 4TMP as well as further analyzed by publicly available AlphaFold tool.

E. Colony formation assay showing effect of re-expression of indicated AF9 proteins ((AF9(WT) and AF9(43-60 aaΔ)) in stable AF9 KD cells on overall colony forming potential. Parallel use of empty vector (EV) was used as control in our experimental setup (n=3 replicates).

F. Cell proliferation assay showing effect of re-expression of indicated AF9 proteins ((AF9(WT) and AF9(43-60 aa Δ)) in stable AF9 KD cells on overall proliferation ability (n=3 replicates).

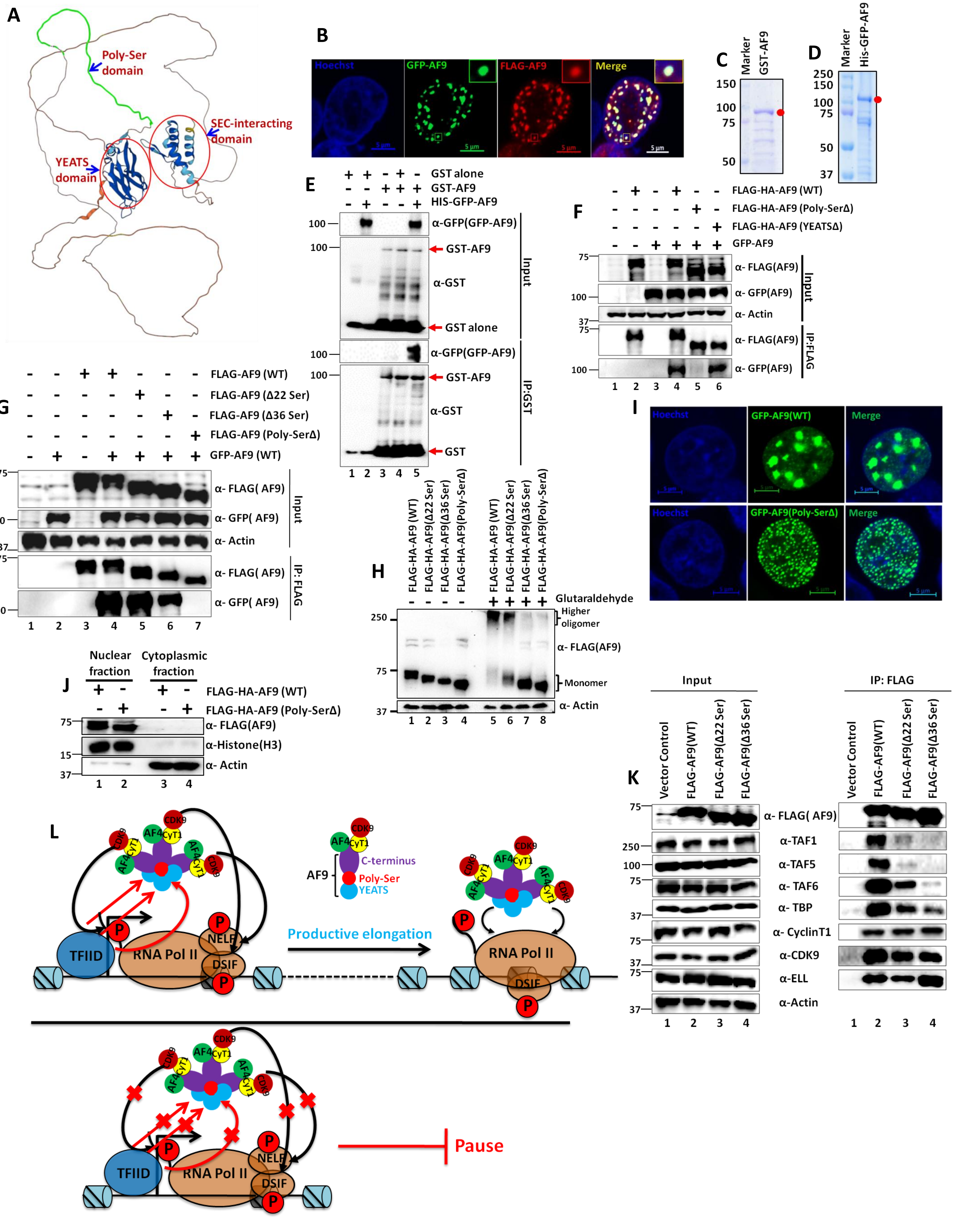
A
Poly-Ser domain
SEC-interacting domain
YEATS domain
B
Hoechst
GFP-AF9
FLAG-AF9
Merge
5 µm
C
Marker
GST-AF9
150
100
75
50
D
Marker
His-GFP-AF9
250
150
100
75
50
37
E
GST alone
GST-AF9
HIS-GFP-AF9
α-GFP(GFP-AF9)
GST-AF9
α-GST
GST alone
Input
GST
IP:GST
1 2 3 4 5
F
FLAG-HA-AF9 (WT)
FLAG-HA-AF9 (Poly-SerΔ)
FLAG-HA-AF9 (YEATSΔ)
GFP-AF9
α- FLAG(AF9)
α- GFP(AF9)
α- Actin
Input
IP:FLAG
1 2 3 4 5 6
G
FLAG-AF9 (WT)
FLAG-AF9 (Δ22 Ser)
FLAG-AF9 (Δ36 Ser)
FLAG-AF9 (Poly-SerΔ)
GFP-AF9 (WT)
α- FLAG(AF9)
α- GFP(AF9)
α- Actin
Input
IP: FLAG
1 2 3 4 5 6 7
H
FLAG-HA-AF9 (WT)
FLAG-HA-AF9(Δ22 Ser)
FLAG-HA-AF9(Δ36 Ser)
FLAG-HA-AF9(Poly-SerΔ)
Glutaraldehyde
Higher oligomer
α- FLAG(AF9)
Monomer
α- Actin
1 2 3 4 5 6 7 8
I
Hoechst
GFP-AF9(WT)
GFP-AF9(Poly-SerΔ)
Merge
J
Nuclear fraction
Cytoplasmic fraction
FLAG-HA-AF9 (WT)
FLAG-HA-AF9 (Poly-SerΔ)
α- FLAG(AF9)
α-Histone(H3)
α- Actin
1 2 3 4
K
Input
IP: FLAG
Vector Control
FLAG-AF9(WT)
FLAG-AF9(Δ22 Ser)
FLAG-AF9(Δ36 Ser)
α- FLAG(AF9)
α-TAF1
α-TAF5
α- TAF6
α- TBP
α- CyclinT1
α-CDK9
α-ELL
α-Actin
1 2 3 4
L
CDK9
AF4
CyT1
AF9
C-terminus
Poly-Ser
YEATS
TFIID
RNA Pol II
NELF
DSIF
P
Productive elongation
Pause

Supplementary Figure 2: Poly-Ser domain-dependent self-association and oligomerization is important for AF9-mediated target gene activation within 293T cells.

A. Predicted structural features of the full-length human AF9 protein as obtained through AlphaFold tool. As can be seen, both the N-terminal YEATS domain and C-terminal SEC-interacting domains form closely juxtaposed interacting surface with structural domains. The remaining AF9 sequences do not predict to form any structural surface and rather form long stretch of loop region which presumably could regulate cognate interactions with their associated structural YEATS and SEC-interacting domains. Also, important to note that the Poly-Ser domain forms a loop region close to the YEATS domain.

B. Immunofluorescence analysis showing self-association between ectopically-expressed FLAG-HA-AF9 and GFP-AF9 within mammalian cells by confocal microscopy (n=3 replicates).

C-D. SDS-PAGE Coomassie staining of purified recombinant full-length GST-AF9 (n=2 replicates) and His-GFP-AF9 (n=3 replicates), as indicated.

E. Immunoblotting analysis showing self-association between purified full-length GST-AF9 and His-GFP-AF9 proteins at physiological salt concentration (150mM NaCl) *in vitro* (n=3 replicates).

F. Immunoblotting analysis showing Poly-Ser domain, and not the YEATS domain, is crucial for self-association of AF9 (n=2 replicates).

G. Immunoblotting analysis showing that strength of self-association is dependent on the stretch of the serine residues present within AF9 (n=2 replicates).

H. Immunoblotting analysis showing oligomerization of ectopically-expressed FLAG-HA-AF9 proteins containing indicated fragments (WT, Δ22 Ser, Δ36Ser and Poly-SerΔ) (n=2 replicates).

I. Immunofluorescence analysis showing presence of both the GFP-AF9(WT) and GFP-AF9(Poly-SerΔ) exclusively within the nucleus of mammalian cells (n=3 replicates).

J. Biochemical fractionation and immunoblotting analysis showing presence of the FLAG-AF9(WT) and FLAG-AF9(Poly-SerΔ) exclusively within the nucleus of mammalian cells (n=1 replicate).

K. Immunoblotting analysis showing interaction of ectopically-expressed FLAG-HA-AF9 proteins (WT, Δ22 Ser, Δ36 Ser) with indicated TFIID and SEC components (n=2 replicates).

L. Model depicting Poly-Ser domain-dependent oligomerization of AF9 potentially brings multiple interacting YEATS domain surfaces for efficient interaction with several specific TAF components within TFIID complex for optimal interaction and transcriptional activation through recruitment of AF9-associated SEC components at the promoter-proximal region for release of paused Pol II through the action of P-TEFb complex. A failure of this oligomerization leads to inefficient interaction and impaired recruitment of SEC components and thus lead to enhanced pausing of Pol II at the target genes.

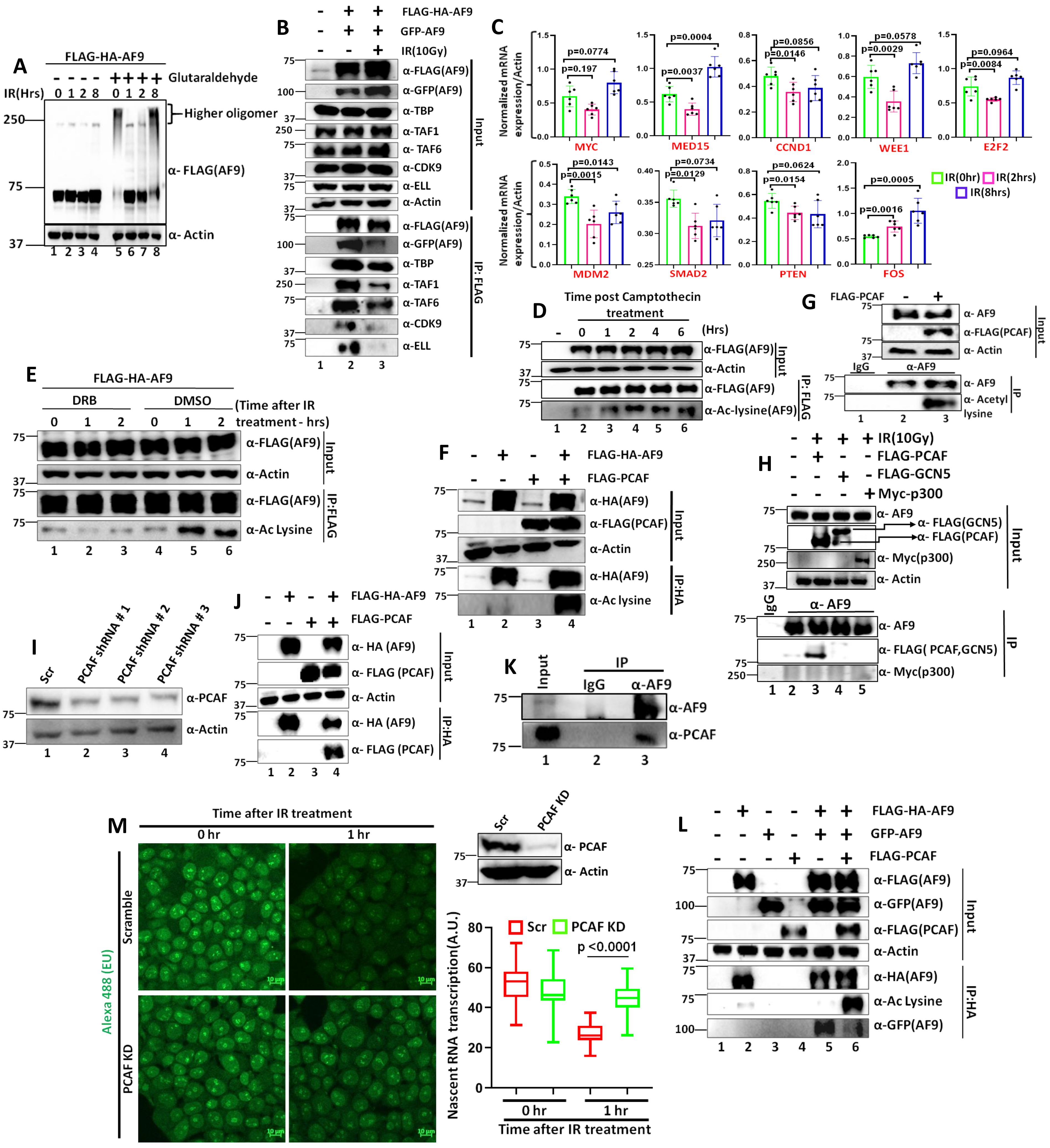

Supplementary Figure 3: PCAF-mediated acetylation leading to reduced self-association of AF9 coincides with genotoxic stress-dependent reduced TFIID interaction and global transcriptional downregulation within 293T cells.

A. Immunoblotting analysis showing dynamic oligomerization of ectopically-expressed FLAG-HA-AF9 protein (n=2 replicates).

B. Immunoblotting analysis showing IR-induced reduced self-association between ectopically-expressed FLAG-HA-AF9(WT) and GFP-AF9(WT) proteins within mammalian cells and its subsequent effect on TFIID and SEC interaction (n=3 replicates).

C. qRT-PCR analysis showing dynamic effect of IR-induced genotoxic stress on mRNA expression of AF9-target genes as mentioned (n=2 replicates).

D. Immunoblotting analysis showing acetylation of ectopically-expressed AF9 protein upon exposure to Camptothecin (5µM) at indicated time points (n=1 replicate).

E. Immunoblotting analysis showing the effect of transcription inhibition on genotoxic stress-induced acetylation of AF9. 293T cells, ectopically-expressing FLAG-HA-AF9, were treated with DMSO or DRB at 50μM final concentration prior to IR(10Gy) treatment for addressing the overall effect of transcription inhibition on AF9 acetylation (n=2 replicates).
F. Immunoblotting analysis showing acetylation of ectopically-expressed AF9 by PCAF (n=3 replicates).
G. Immunoblotting analysis showing acetylation of endogenous AF9 upon ectopic expression of FLAG-PCAF within mammalian cells (n=1 replicate).
H. Immunoblotting analysis showing specific interaction of endogenous AF9 protein with ectopically-expressed PCAF only in presence of IR treatment (n=1 replicate).
I. Immunoblotting analysis showing stable knockdown (KD) of endogenous PCAF protein by multiple shRNAs (n=3 replicates).
J. Immunoblotting analysis showing interaction of AF9 with PCAF within mammalian cells (n=2 replicates).
K. Immunoblotting analysis showing interaction of endogenous AF9 with PCAF within mammalian cells (n=1 replicate).
L. Immunoblotting analysis showing effect of PCAF-mediated acetylation on self-association between ectopically-expressed FLAG-HA-AF9(WT) and GFP-AF9(WT) proteins within mammalian cells (n=2 replicates).
M. Nascent RNA transcription analysis (through EU incorporation) showing effect of PCAF KD on global transcriptional downregulation after IR(10Gy) treatment. Stable PCAF KD cells were treated with one time IR(10Gy). 1hr after IR treatment, cells were subjected to EU incorporation assay for addressing the overall transcription potential within cells. The boxes represent median and quartiles and value ranges of 25-75 and 10-90%. Upper and lower hinges extend to largest and smallest data points (n=100 cells). This experiment was done once.

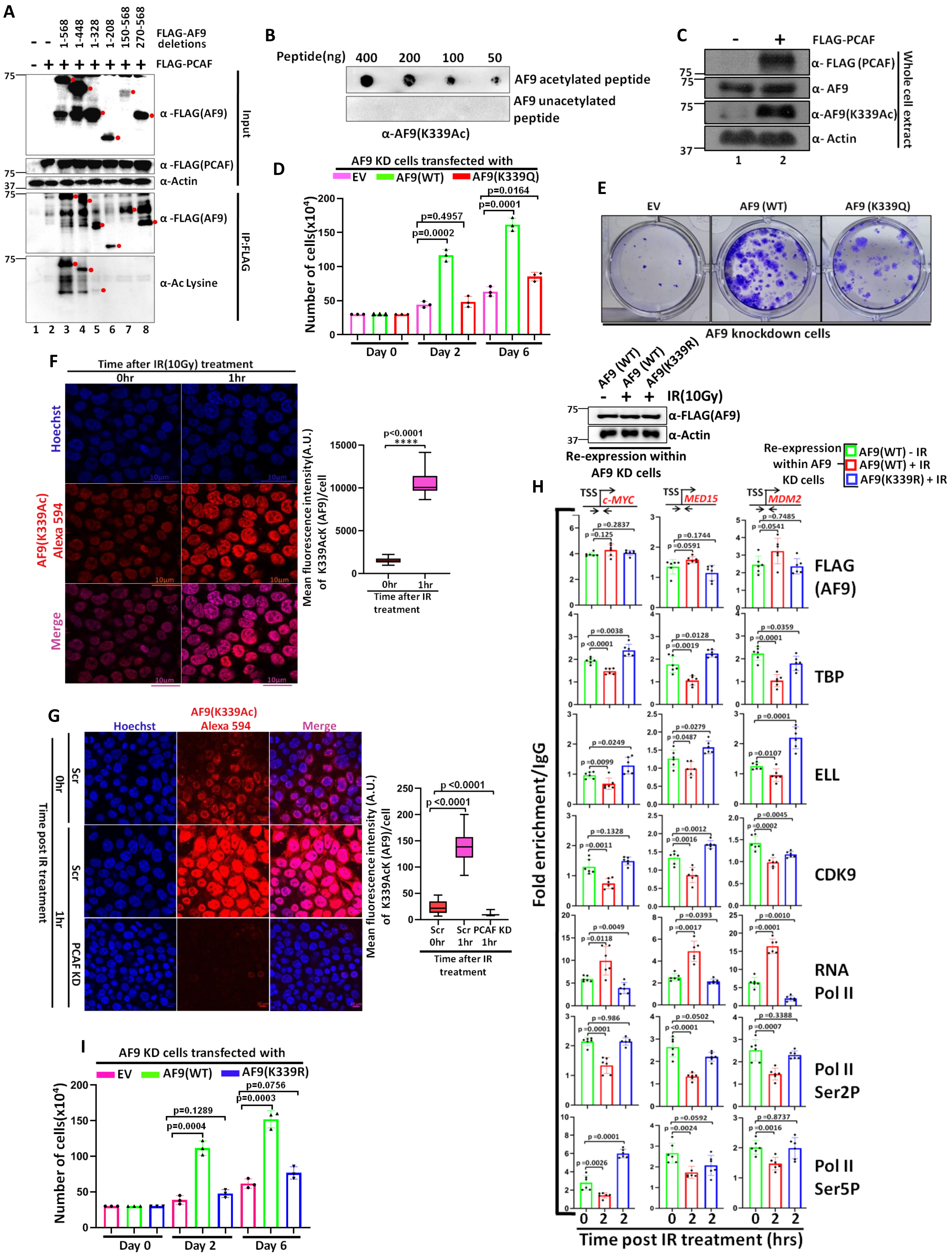

A
FLAG-AF9 deletions
1-568
1-448
1-328
1-208
150-568
270-568
FLAG-PCAF
α -FLAG(AF9)
α -FLAG(PCAF)
α-Actin
α -FLAG(AF9)
α-Ac Lysine
Input
IP:FLAG
1 2 3 4 5 6 7 8
B
Peptide(ng) 400 200 100 50
AF9 acetylated peptide
AF9 unacetylated peptide
α-AF9(K339Ac)
C
FLAG-PCAF
α- FLAG (PCAF)
α- AF9
α-AF9(K339Ac)
α- Actin
Whole cell extract
1 2
D
AF9 KD cells transfected with
EV
AF9(WT)
AF9(K339Q)
Number of cells(x10^4)
p=0.4957
p=0.0002
p=0.0164
p=0.0001
Day 0
Day 2
Day 6
E
EV
AF9 (WT)
AF9 (K339Q)
AF9 knockdown cells
F
Time after IR(10Gy) treatment
0hr
1hr
Hoechst
AF9(K339Ac) Alexa 594
Merge
10μm
p<0.0001
****
Mean fluorescence intensity(A.U.) of K339AcK (AF9)/cell
Time after IR treatment
AF9 (WT)
AF9 (WT)
AF9(K339R)
IR(10Gy)
α-FLAG(AF9)
α-Actin
Re-expression within AF9 KD cells
G
Hoechst
AF9(K339Ac) Alexa 594
Merge
Scr
PCAF KD
0hr
1hr
Time post IR treatment
p <0.0001
p <0.0001
Scr 0hr
Scr 1hr
PCAF KD 1hr
Time after IR treatment
Re-expression within AF9 KD cells
AF9(WT) - IR
AF9(WT) + IR
AF9(K339R) + IR
H
TSS
c-MYC
MED15
MDM2
FLAG (AF9)
TBP
ELL
CDK9
RNA Pol II
Pol II Ser2P
Pol II Ser5P
Fold enrichment/IgG
Time post IR treatment (hrs)
0 2 2
I
AF9 KD cells transfected with
EV
AF9(WT)
AF9(K339R)
Number of cells(x10^4)
p=0.1289
p=0.0004
p=0.0756
p=0.0003
Day 0
Day 2
Day 6

Supplementary Figure 4: Acetylation of AF9 at K339 residue is important for global transcriptional downregulation upon genotoxic stress within 293T cells.

A. Immunoblotting analysis showing acetylation of different domains of AF9 by PCAF within mammalian cells (n=2 replicates).

B. Dot blot assay showing specificity of raised polyclonal antibody (AF9(K339AcK) for detection of modified AF9 acetylated peptides only (n=2 replicates).

C. Immunoblotting analysis showing acetylation of endogenous AF9 at K339 residue upon ectopic expression of PCAF within 293T cells (n=2 replicates).

D. Cell proliferation assay showing the effect of re-expression of indicated AF9 proteins ((AF9(WT) and AF9(K339Q)) in stable AF9 KD cells on overall proliferation ability (n=3 replicates).

E. Colony formation assay showing the effect of re-expression of indicated AF9 proteins ((AF9(WT) and AF9(K339Q)) in stable AF9 KD cells on overall colony formation potential (n=3 replicates).

F. Immunofluorescence analysis showing enhanced acetylation of endogenous AF9 at K339 residue at 1hr time point post IR(10Gy) treatment within 293T cells by confocal microscopy. The boxes represent median and quartiles and value ranges of 25-75 and 10-90%. Upper and lower hinges extend to largest and smallest data points (n=57 cells). This experiment was done once.

G. Immunofluorescence assay showing effect of PCAF KD on acetylation of endogenous AF9 at K339 residue by confocal microscopy. Presence of AF9(K339Ac) was checked by immunofluorescence study using specific antibody. The boxes represent median and quartiles and value ranges of 25-75 and 10-90%. Upper and lower hinges extend to largest and smallest data points (n=49 cells). This experiment was done once.

H. ChIP analysis showing effect of re-expression of AF9(WT) and AF9(K339R) proteins in stable AF9 KD cells on recruitment of indicated target factors at the promoter-proximal region of selected AF9 target genes at 2hrs time point post IR(10Gy) treatment. Data represents a minimum of n=2 biological replicates and a minimum of three PCR replicates for each biological replicate. The error bar represents mean ± SD and statistical analyses were performed using one-tailed Student's *t* test. *p* values for each experimental data is mentioned on the bar diagram.

I. Cell proliferation assay showing the effect of re-expression of indicated AF9 proteins ((AF9(WT) and AF9(K339Q)) in stable AF9 KD cells on overall proliferation ability after treatment with IR(10Gy) (n=2 replicates).

A

Name of protein	Percentage coverage	Total no. of peptides	Total no. of unique peptides	Representative peptides
DNA-PKc	11	68	39	HGDLPDIQIK
DNA-PKc	11	68	39	TVGALQVLGTEAQSSLLK
DNA-PKc	11	68	39	VTELALTASDR
DNA-PKc	11	68	39	NLSSNEAISLEEIR
DNA-PKc	11	68	39	ATQQQHDFTLTQTADGR
DNA-PKc	11	68	39	LNESTFDTQITK
DNA-PKc	11	68	39	VVQMLGSLGGQINK
DNA-PKc	11	68	39	AQEPESGLSEETQVK
DNA-PKc	11	68	39	SQGCSEQVLTVLK
DNA-PKc	11	68	39	INQVFHGSCITEGNELTK
DNA-PKc	11	68	39	NNWEVSALSR
DNA-PKc	11	68	39	LGLPGDEVDNK
DNA-PKc	11	68	39	NELEIPGQYDGR

B

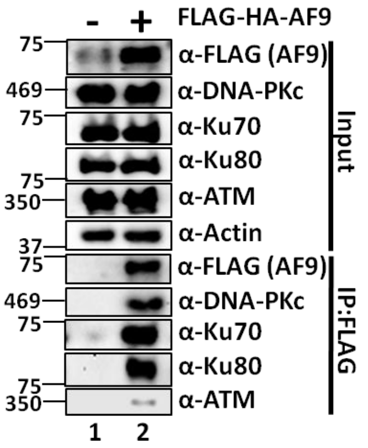

C

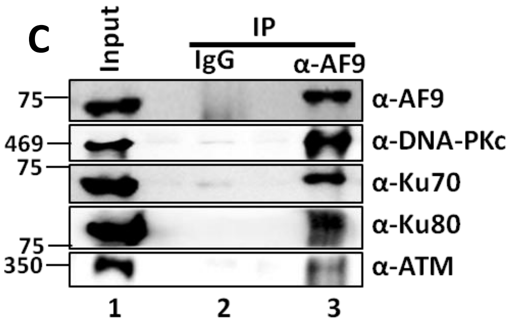

Supplementary Figure 5: DNA-PKc is a novel interactor of AF9 within 293T cells.

A. Mass Spectrometric analysis showing representative peptides of DNA-PKc in the AF9-immunoprecipitated sample and thus indicating its interaction with ectopically-expressed AF9 within mammalian 293T cells. This experiment was done once.

B. Immunoblotting analysis showing specific interaction of ectopically-expressed AF9 protein with endogenous DNA-PKc only within mammalian cells. This experiment was done once.

C. Immunoblotting analysis showing specific interaction of AF9 protein with DNA-PKc only in endogenous context within mammalian cells. This experiment was done once.

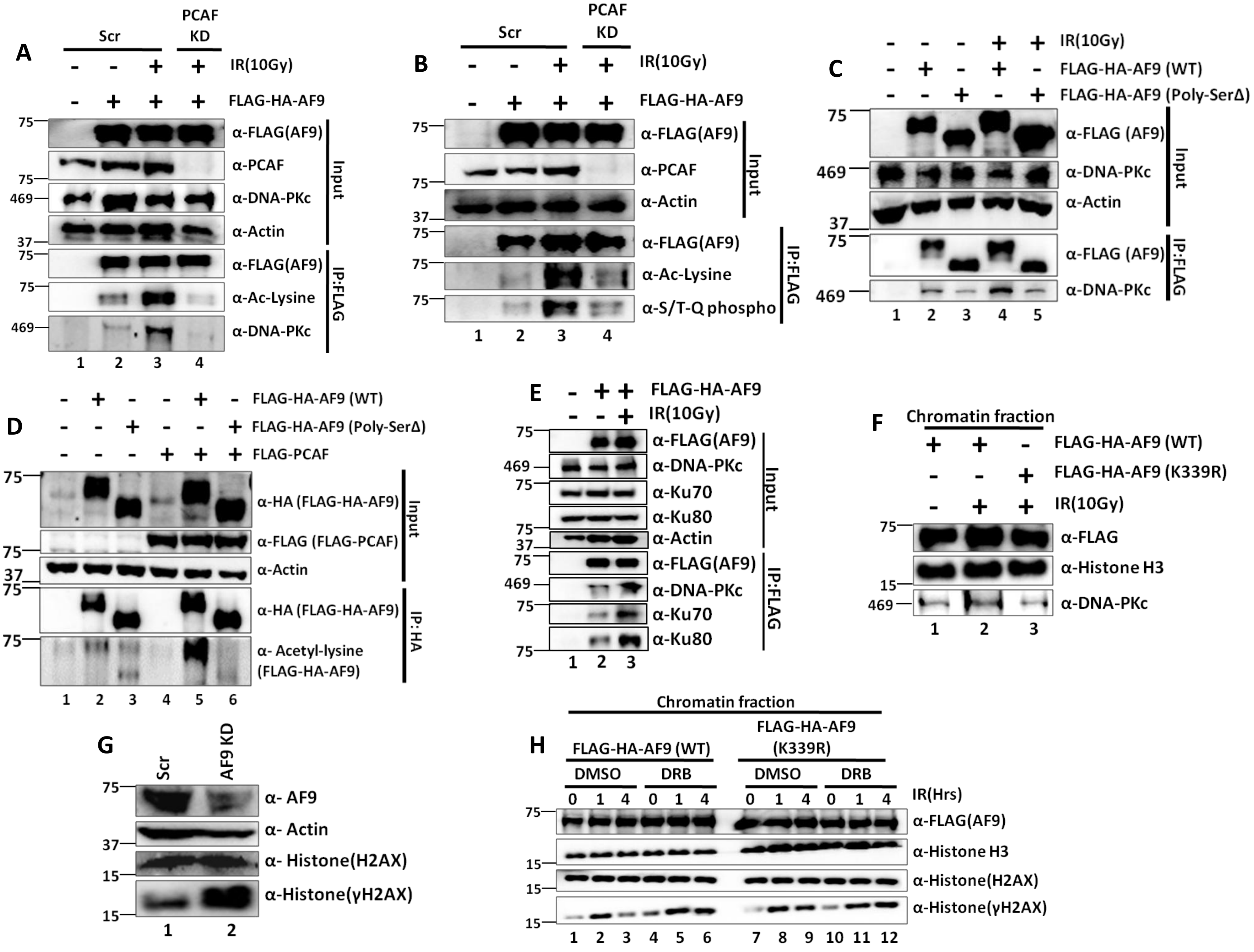

Supplementary Figure 6: Genotoxic stress-induced AF9 acetylation-dependent recruitment of DNA-PKc onto chromatin for DNA damage repair within 293T cells.

A. Immunoblotting analysis showing effect of PCAF KD on IR-induced acetylation of ectopic AF9 and its concomitant interaction with DNA-PKc within mammalian cells (n=2 replicates).

B. Immunoblotting analysis showing effect of PCAF KD on IR treatment-dependent acetylation and phosphorylation of ectopic AF9 within mammalian cells (n=2 replicates).

C. Immunoblotting analysis showing effect of monomer formation of AF9 on its IR treatment-dependent enhanced interaction with DNA-PKc (n=2 replicates).

D. Immunoblotting analysis showing effect of Poly-Ser deletion of AF9 on its acetylation by PCAF within mammalian cells (n=2 replicates).

E. Immunoblotting analysis showing IR(10Gy) treatment-dependent enhanced interaction of ectopically-expressed AF9 with DNA-PKc and concomitant Ku complex components within mammalian cells (n=2 replicates).

F. Immunoblotting analysis showing the effect of acetylation-defective AF9 mutant (K339R) on IR treatment-dependent recruitment of DNA-PKc as well as Ku complex components onto the chromatin within mammalian cells (n=1 replicate).

G. Immunoblot analysis showing presence of enhanced level of DNA damage in AF9 knockdown cells even under normal cellular growth as assessed through western blotting analysis using γ-H2AX antibody (n=2 replicates).

H. Immunoblotting analysis showing the effect of transcription inhibition on repair of damaged DNA at indicated time points after one-time IR(10Gy) treatment. 293T cells, transfected with indicated plasmids, were exposed to IR(10Gy) and treated with DMSO or DRB at 50µM final concentration immediately within 10min after IR treatment and chromatin fractions were prepared after indicated time points of IR treatment (n=1 replicate).

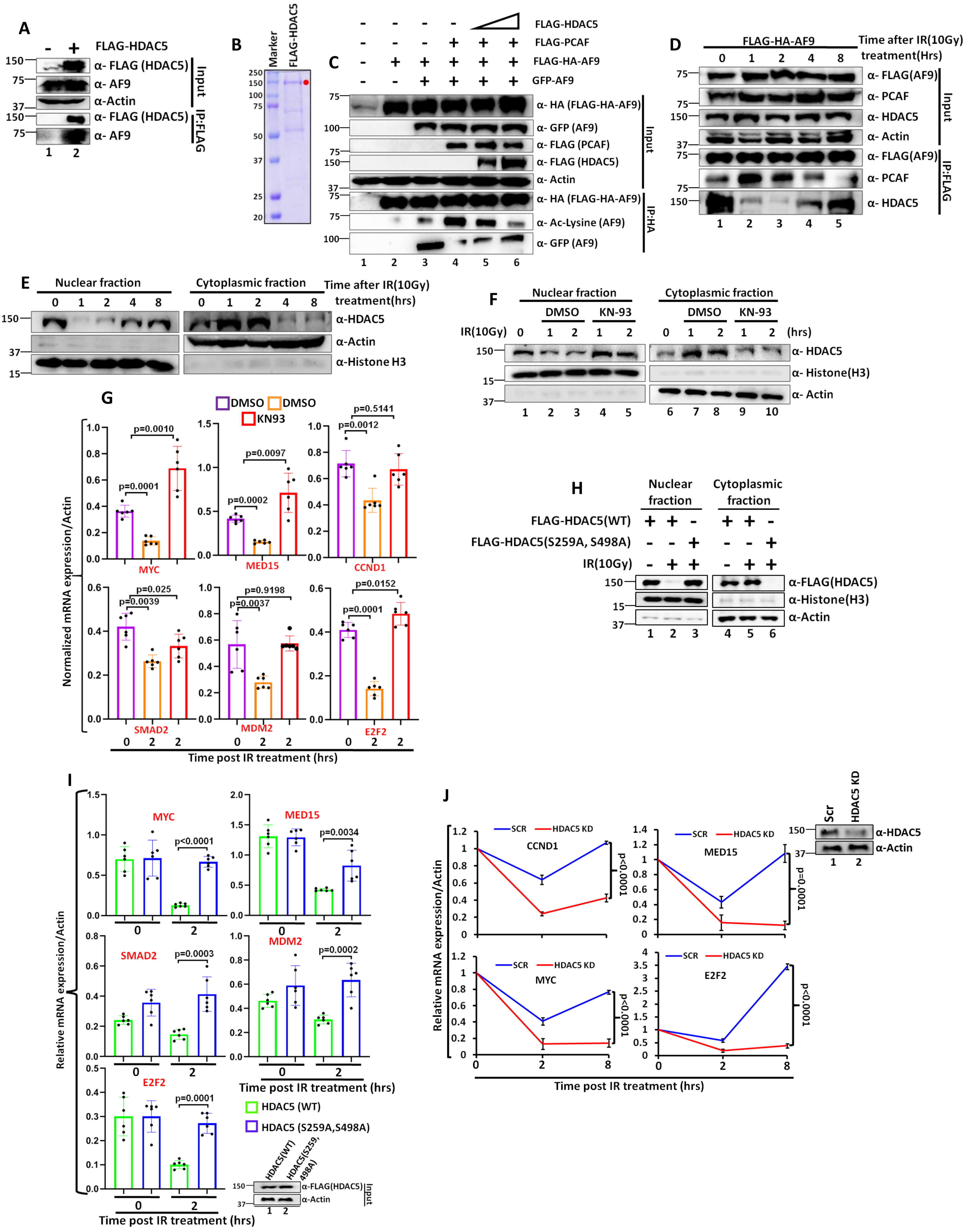

A
FLAG-HDAC5
α- FLAG (HDAC5)
α- AF9
α-Actin
Input
IP:FLAG
B
Marker
FLAG-HDAC5
C
FLAG-HDAC5
FLAG-PCAF
FLAG-HA-AF9
GFP-AF9
α- HA (FLAG-HA-AF9)
α- GFP (AF9)
α- FLAG (PCAF)
α- FLAG (HDAC5)
α- Actin
α- Ac-Lysine (AF9)
IP:HA
D
FLAG-HA-AF9
Time after IR(10Gy) treatment(Hrs)
α- FLAG(AF9)
α- PCAF
α- HDAC5
α- Actin
E
Nuclear fraction
Cytoplasmic fraction
Time after IR(10Gy) treatment(hrs)
α-HDAC5
α-Actin
α-Histone H3
F
Nuclear fraction
Cytoplasmic fraction
DMSO
KN-93
IR(10Gy)
(hrs)
α- HDAC5
α- Histone(H3)
α- Actin
G
DMSO
DMSO
KN93
Normalized mRNA expression/Actin
MYC
MED15
CCND1
SMAD2
MDM2
E2F2
p=0.0010
p=0.0001
p=0.0097
p=0.0002
p=0.5141
p=0.0012
p=0.025
p=0.0039
p=0.9198
p=0.0037
p=0.0152
p=0.0001
Time post IR treatment (hrs)
H
Nuclear fraction
Cytoplasmic fraction
FLAG-HDAC5(WT)
FLAG-HDAC5(S259A, S498A)
IR(10Gy)
α-FLAG(HDAC5)
α-Histone(H3)
α-Actin
I
Relative mRNA expression/Actin
MYC
MED15
SMAD2
MDM2
E2F2
p<0.0001
p=0.0034
p=0.0003
p=0.0002
p=0.0001
Time post IR treatment (hrs)
HDAC5 (WT)
HDAC5 (S259A,S498A)
HDAC5(WT)
HDAC5(S259, 498A)
α-FLAG(HDAC5)
α-Actin
Input
J
Relative mRNA expression/Actin
SCR
HDAC5 KD
CCND1
MED15
MYC
E2F2
p<0.0001
Scr
HDAC5 KD
α-HDAC5
α-Actin
Time post IR treatment (hrs)

Supplementary Figure 7: Genotoxic stress-dependent nuclear and cytoplasmic localization of HDAC5 is crucial for temporal regulation of transcription within 293T cells.
IA. Immunoblotting analysis showing interaction of ectopically-expressed HDAC5 with endogenous AF9 (n=2 replicates).
B. SDS-PAGE Coomassie staining of purified full-length FLAG-HDAC5 from mammalian 293T cells (n=2 replicates).
C. Immunoblot analysis showing opposite effect of PCAF and HDAC5 on AF9 self-association within mammalian cells (n=2 replicates).
D. Immunoblotting analysis showing the dynamic interaction of ectopically-expressed AF9 with endogenous PCAF and HDAC5 within mammalian cells upon exposure to genotoxic stress (n=3 replicates).
E. Immunoblotting analysis showing genotoxic stress-dependent nuclear cytoplasmic shuttling of endogenous HDAC5. Both nuclear and cytoplasmic fractions were prepared from cells treated with one time IR (10Gy). Subsequent western blotting analysis using HDAC5-specific antibody was performed to address the presence of HDAC5 in the target fractions (n=2 replicates).
F. Immunoblotting analysis showing effect of prior treatment with CaMKII inhibitor (KN-93) on IR-dependent nuclear cytoplasmic shuttling of endogenous HDAC5. 293T cells were treated with DMSO or KN-93 at 10µM final concentration and were subjected to one time IR (10Gy). Nuclear and cytoplasmic fractions prepared from these cells were subjected to western blotting analysis with indicated antibodies for testing the overall effect (n=1 replicate).
G. qRT-PCR analysis showing the effect of CaMKII inhibitor (KN-93) on mRNA expression of indicated AF9 target genes at 2hrs post IR(10Gy) treatment (n=2 replicates).
H. Immunoblotting analysis showing effect of IR(10Gy) treatment on nuclear cytoplasmic shuttling of CaMKII-mediated phosphorylation-defective HDAC5 mutant (S259A, S498A) within mammalian cells (n=1 replicate).
I. qRT-PCR analysis showing effect of ectopic expression of HDAC5(WT) and HDAC5(S259A,498A) proteins on mRNA expression of indicated AF9 target genes within mammalian cells at 2hrs after exposure to IR (n=2 replicates).
J. mRNA expression analysis of AF9 target genes by qRT-PCR showed defective transcriptional restart at 8hrs time point in the HDAC5 KD cells when compared to control Scr cells (n=2 replicates).
For this figure, the data showing RNA analyses by qRT-PCR, the error bar represents mean ± SD and statistical analyses were performed using one-tailed Student's *t* test. *p* values for each experimental data is mentioned on the bar diagram. Data represents a minimum of n=2 biological replicates and three PCR replicates for each biological replicate.

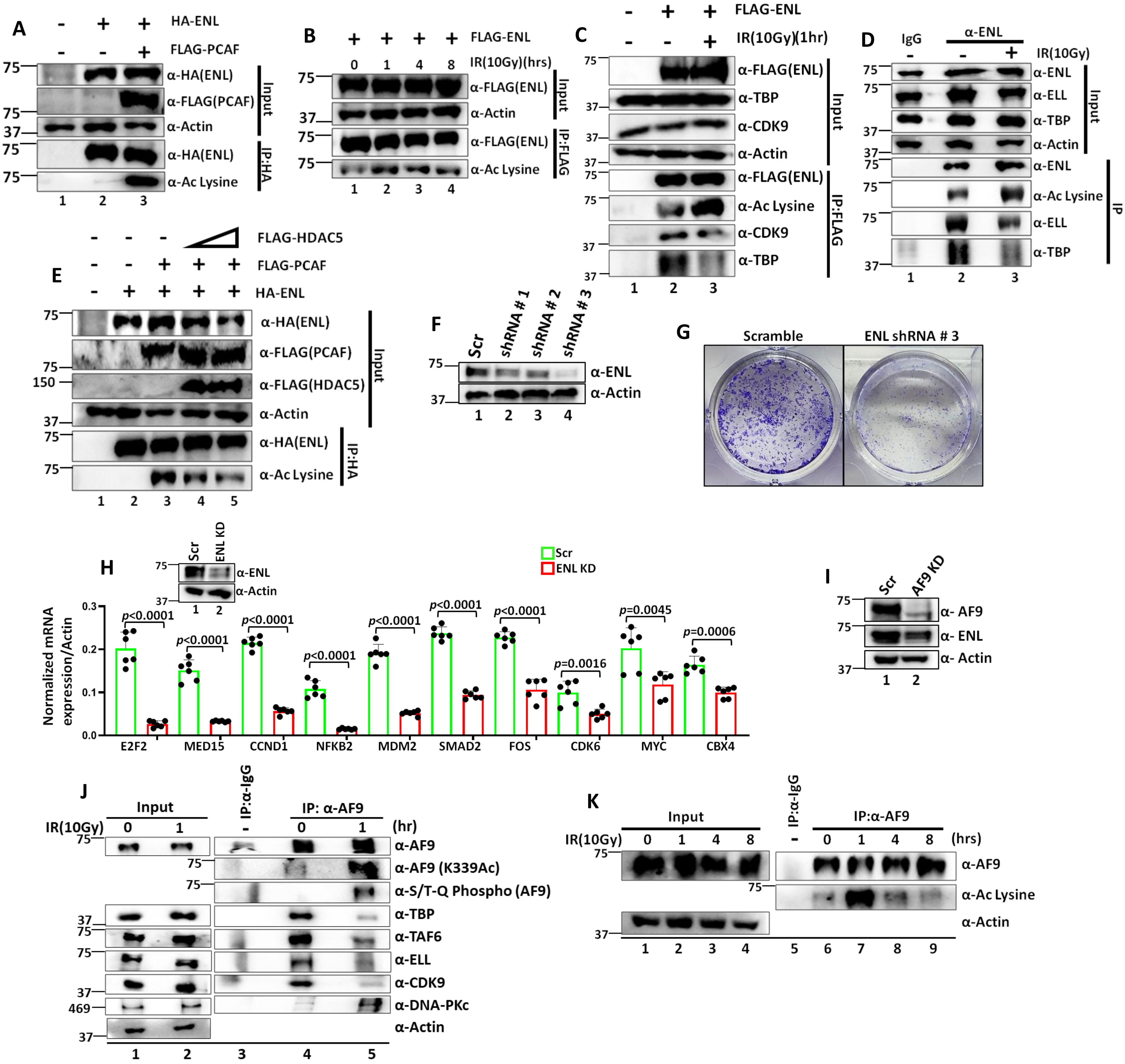

Supplementary Figure 8: Like AF9, other AF9 family protein, ENL is also subjected to similar mechanism of regulation within 293T as well as HeLa cells.
A. Immunoblotting analysis showing acetylation of ENL by PCAF within mammalian cells (n=2 replicates).
B. Immunoblotting analysis showing dynamic acetylation of ectopically-expressed ENL protein upon exposure to IR(10Gy) (n=2 replicates).
C. Immunoblotting analysis showing effect of IR(10Gy) treatment on acetylation and concomitant interaction of TFIID and SEC components with ectopic ENL protein (n=2 replicates).
D. Immunoblotting analysis showing effect of ionizing radiation treatment on acetylation and concomitant interaction of TFIID and SEC components with endogenous ENL protein (n=1 replicate).
E. Immunoblotting analysis showing deacetylation of ENL by concomitant expression of HDAC5 within 293T cells (n=1 replicate).
F. Immunoblotting analysis showing stable knockdown of ENL protein by multiple shRNAs as indicated (n=3 replicates).
G. Colony formation assay showing effect of stable knockdown of ENL on overall colony formation ability of 293T cells (n=2 replicates).
H. qRT-PCR analysis showing the effect of ENL knockdown on expression of indicated target genes within mammalian cells (n=2 replicates).
I. Immunoblotting analysis showing effect of AF9 knockdown on expression of ENL protein within mammalian 293T cells (n=1 replicate).
J. Immunoblotting analysis showing genotoxic stress-dependent acetylation of AF9 within HeLa cells (n=1 replicate).
K. Immunoblotting analysis showing genotoxic stress-dependent acetylation-mediated reduced interaction of endogenous AF9 with TFIID and SEC components and concomitant enhanced interaction with DNA-PKc in HeLa cells (n=1 replicate).

Supplementary Figure 9

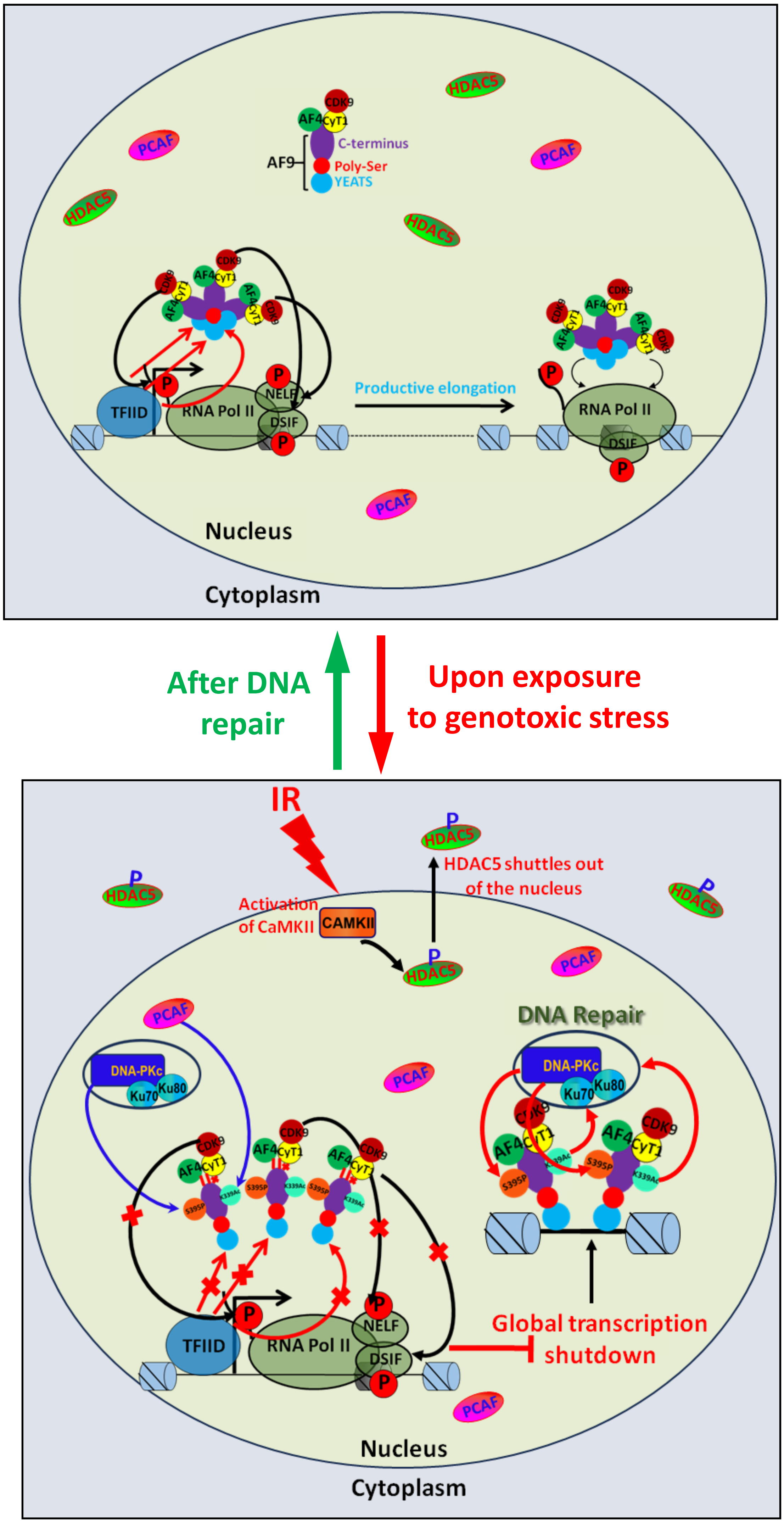

Supplementary Figure 9: Overall model of action for regulation of global transcription as well as DNA repair during genotoxic stress involving AF9. The cartoon diagram depicts overall mechanisms of action of regulating transcription and DNA repair during genotoxic stress through involvement of functional regulation of AF9 by post-translational modifications by associated factors. Under normal condition of cellular growth, Poly-Ser domain-dependent oligomerization of AF9 protein leads to its efficient interaction with TFIID complex for its recruitment at the promoter-proximal region for the downstream recruitment of SEC components for aiding release of paused Pol II. In absence of this recruitment, the release of paused Pol II does not happen and transcriptional activation is impaired.

Upon exposure to genotoxic stress, our study deciphered a novel regulatory pathway for global transcriptional downregulation within mammalian cells that involves regulation of oligomerization potential of AF9 (and likely other family member protein ENL as well). After exposure to genotoxic stress, enhanced ROS generation leads to activation of CaMKII that in turn phosphorylates class II histone deacetylases including HDAC5. Upon phosphorylation, HDAC5 is shuttled out of nucleus to cytoplasm leading to enhanced interaction of nuclear AF9 with specific acetyl transferase PCAF. This enhanced interaction causes PCAF-mediated acetylation of AF9 at key K339 residue that results in monomerization of the protein. The monomerized AF9 loses its interaction with TFIID complex and thus causes initial global transcriptional downregulation. The acetylation of AF9 at K339 residue further causes its enhanced interaction with DNA-PKc and thus leads to its phosphorylation at S395 residue and loss of interaction with SEC components and optimal global transcriptional downregulation. AF9 acetylation (K339 residue)-mediated enhanced recruitment of DNA-PKc onto chromatin further recruits Ku complex for repairing of damaged DNA that involves NHEJ pathway. After repair of damaged DNA, HDAC5 is shuttled back to nucleus leading to deacetylation of AF9 and restoration of its oligomerization potential for transcriptional activation. Restoration of this transcriptional activation is absolutely essential for the survival of the cell after repair of damaged DNA.
