# Supplementary material for: Post-translational modification-dependent oligomerization switch in regulation of global transcription and DNA damage repair during genotoxic stress
Source: Nat Commun. 2024 May 15;15:4128. doi: 10.1038/s41467-024-48530-8 (PMC11096357; doi:10.1038/s41467-024-48530-8)
Supplement: Supplementary file 3 — Description of Additional Supplementary Files [file 41467_2024_48530_MOESM3_ESM.pdf]

Description of Additional Supplementary Files

File Name: Supplementary Data 1
Description: List of plasmids used in this study.

File Name: Supplementary Data 2
Description: List of antibodies used in this study.

File Name: Supplementary Data 3
Description: List of qRT-PCR primers used for RNA and ChIP analysis in this study.

File Name: Supplementary Data 4
Description: List of oligo sequences for shRNA construct generation for AF9, HDAC5, PCAF and ENL knockdown.

File Name: Supplementary Data 5
Description: List of all reagents used in this paper including antibodies.
