# Supplementary material for: Post-translational modification-dependent oligomerization switch in regulation of global transcription and DNA damage repair during genotoxic stress
Source: Nat Commun. 2024 May 15;15:4128. doi: 10.1038/s41467-024-48530-8 (PMC11096357; doi:10.1038/s41467-024-48530-8)
Supplement: Supplementary file 5 — Reporting Summary [file 41467_2024_48530_MOESM5_ESM.pdf]

nature portfolio

Corresponding author(s): Debabrata Biswas

Last updated by author(s): Apr 6, 2024

Reporting Summary

Nature Portfolio wishes to improve the reproducibility of the work that we publish. This form provides structure for consistency and transparency in reporting. For further information on Nature Portfolio policies, see our Editorial Policies and the Editorial Policy Checklist.

Statistics

For all statistical analyses, confirm that the following items are present in the figure legend, table legend, main text, or Methods section.

n/a	Confirmed	
☐	☒	The exact sample size (*n*) for each experimental group/condition, given as a discrete number and unit of measurement
☐	☒	A statement on whether measurements were taken from distinct samples or whether the same sample was measured repeatedly
☐	☒	The statistical test(s) used AND whether they are one- or two-sided<br>*Only common tests should be described solely by name; describe more complex techniques in the Methods section.*
☒	☐	A description of all covariates tested
☒	☐	A description of any assumptions or corrections, such as tests of normality and adjustment for multiple comparisons
☐	☒	A full description of the statistical parameters including central tendency (e.g. means) or other basic estimates (e.g. regression coefficient) AND variation (e.g. standard deviation) or associated estimates of uncertainty (e.g. confidence intervals)
☐	☒	For null hypothesis testing, the test statistic (e.g. *F*, *t*, *r*) with confidence intervals, effect sizes, degrees of freedom and *P* value noted<br>*Give P values as exact values whenever suitable.*
☒	☐	For Bayesian analysis, information on the choice of priors and Markov chain Monte Carlo settings
☒	☐	For hierarchical and complex designs, identification of the appropriate level for tests and full reporting of outcomes
☒	☐	Estimates of effect sizes (e.g. Cohen's *d*, Pearson's *r*), indicating how they were calculated

Our web collection on statistics for biologists contains articles on many of the points above.

Software and code

Policy information about availability of computer code

Data collection	No software was used for data collection for our study.
Data analysis	For all of our RNA and ChIP analysis, data were analyzed by using GraphPad Prism software (v8.0.2). LSM 800 (ZEISS) confocal microscope was used for imaging and the obtained images were subsequently analyzed using Zen 2.3lite software.

For manuscripts utilizing custom algorithms or software that are central to the research but not yet described in published literature, software must be made available to editors and reviewers. We strongly encourage code deposition in a community repository (e.g. GitHub). See the Nature Portfolio guidelines for submitting code & software for further information.

Data

Policy information about availability of data

All manuscripts must include a data availability statement. This statement should provide the following information, where applicable:

- Accession codes, unique identifiers, or web links for publicly available datasets
- A description of any restrictions on data availability
- For clinical datasets or third party data, please ensure that the statement adheres to our policy

All the original images for western blotting, microscopy analyses that were used for making the figures as described in this study, is available through Source file accompanying with this paper. The datasets for ChIP, RNA and nascent RNA transcription analyses are also available through the Source file . The mass spectrometry analyses for identification of AF9-interacting proteins are available through public repository Mendeley database by clicking the link https://data.mendeley.com/datasets/d2s63vgnmk/1. The original mass spectrometry spectra file is available via ProteomeXchange with identifier PXD049385.

Research involving human participants, their data, or biological material

Policy information about studies with human participants or human data. See also policy information about sex, gender (identity/presentation), and sexual orientation and race, ethnicity and racism.

Reporting on sex and gender	Not applicable
Reporting on race, ethnicity, or other socially relevant groupings	Not applicable
Population characteristics	Not applicable
Recruitment	Not applicable
Ethics oversight	Not applicable

Note that full information on the approval of the study protocol must also be provided in the manuscript.

Field-specific reporting

Please select the one below that is the best fit for your research. If you are not sure, read the appropriate sections before making your selection.

☒ Life sciences ☐ Behavioural & social sciences ☐ Ecological, evolutionary & environmental sciences

For a reference copy of the document with all sections, see nature.com/documents/nr-reporting-summary-flat.pdf

Life sciences study design

All studies must disclose on these points even when the disclosure is negative.

Sample size	No statistical method was employed to predetermine sample size. Cells were randomly chosen for microscopic analyses. For most of our microscopic assays,the number of cells used for analyses are clearly mentioned in the respective figure legends.
Data exclusions	No data exclusion was carried out in this study.
Replication	For RNA and ChIP analysis, a minimum of two(2) independent biological replicates and three(3) PCR replicates of each biological replicate were used. For majority of the key biochemical experiments, minimum of two(2) biological replicates were performed. All the attempts at replication for our RNA, ChIP and other biochemical analyses were successful. No inconsistent results were observed. For individual experiments, the detailed number of replicates have been specified in the respective figure legends.
Randomization	Cells were randomly allocated between control and experimental groups and no biasness was used. All cells were grown in equal conditions as mentioned excepting in the experimental conditions as pertinent for any given experiment and mentioned in the manuscript.
Blinding	Investigators were blinded during sample labelling and group allocation while performing the experiments and data collection.

Behavioural & social sciences study design

All studies must disclose on these points even when the disclosure is negative.

Study description	*Briefly describe the study type including whether data are quantitative, qualitative, or mixed-methods (e.g. qualitative cross-sectional, quantitative experimental, mixed-methods case study).*
Research sample	*State the research sample (e.g. Harvard university undergraduates, villagers in rural India) and provide relevant demographic information (e.g. age, sex) and indicate whether the sample is representative. Provide a rationale for the study sample chosen. For studies involving existing datasets, please describe the dataset and source.*
Sampling strategy	*Describe the sampling procedure (e.g. random, snowball, stratified, convenience). Describe the statistical methods that were used to predetermine sample size OR if no sample-size calculation was performed, describe how sample sizes were chosen and provide a rationale for why these sample sizes are sufficient. For qualitative data, please indicate whether data saturation was considered, and what criteria were used to decide that no further sampling was needed.*
Data collection	*Provide details about the data collection procedure, including the instruments or devices used to record the data (e.g. pen and paper, computer, eye tracker, video or audio equipment) whether anyone was present besides the participant(s) and the researcher, and whether the researcher was blind to experimental condition and/or the study hypothesis during data collection.*
Timing	*Indicate the start and stop dates of data collection. If there is a gap between collection periods, state the dates for each sample cohort.*

Data exclusions	*If no data were excluded from the analyses, state so OR if data were excluded, provide the exact number of exclusions and the rationale behind them, indicating whether exclusion criteria were pre-established.*
Non-participation	*State how many participants dropped out/declined participation and the reason(s) given OR provide response rate OR state that no participants dropped out/declined participation.*
Randomization	*If participants were not allocated into experimental groups, state so OR describe how participants were allocated to groups, and if allocation was not random, describe how covariates were controlled.*

Ecological, evolutionary & environmental sciences study design

All studies must disclose on these points even when the disclosure is negative.

Study description	*Briefly describe the study. For quantitative data include treatment factors and interactions, design structure (e.g. factorial, nested, hierarchical), nature and number of experimental units and replicates.*
Research sample	*Describe the research sample (e.g. a group of tagged Passer domesticus, all Stenocereus thurberi within Organ Pipe Cactus National Monument), and provide a rationale for the sample choice. When relevant, describe the organism taxa, source, sex, age range and any manipulations. State what population the sample is meant to represent when applicable. For studies involving existing datasets, describe the data and its source.*
Sampling strategy	*Note the sampling procedure. Describe the statistical methods that were used to predetermine sample size OR if no sample-size calculation was performed, describe how sample sizes were chosen and provide a rationale for why these sample sizes are sufficient.*
Data collection	*Describe the data collection procedure, including who recorded the data and how.*
Timing and spatial scale	*Indicate the start and stop dates of data collection, noting the frequency and periodicity of sampling and providing a rationale for these choices. If there is a gap between collection periods, state the dates for each sample cohort. Specify the spatial scale from which the data are taken*
Data exclusions	*If no data were excluded from the analyses, state so OR if data were excluded, describe the exclusions and the rationale behind them, indicating whether exclusion criteria were pre-established.*
Reproducibility	*Describe the measures taken to verify the reproducibility of experimental findings. For each experiment, note whether any attempts to repeat the experiment failed OR state that all attempts to repeat the experiment were successful.*
Randomization	*Describe how samples/organisms/participants were allocated into groups. If allocation was not random, describe how covariates were controlled. If this is not relevant to your study, explain why.*
Blinding	*Describe the extent of blinding used during data acquisition and analysis. If blinding was not possible, describe why OR explain why blinding was not relevant to your study.*

Did the study involve field work? ☐ Yes ☐ No

Field work, collection and transport

Field conditions	*Describe the study conditions for field work, providing relevant parameters (e.g. temperature, rainfall).*
Location	*State the location of the sampling or experiment, providing relevant parameters (e.g. latitude and longitude, elevation, water depth).*
Access & import/export	*Describe the efforts you have made to access habitats and to collect and import/export your samples in a responsible manner and in compliance with local, national and international laws, noting any permits that were obtained (give the name of the issuing authority, the date of issue, and any identifying information).*
Disturbance	*Describe any disturbance caused by the study and how it was minimized.*

Reporting for specific materials, systems and methods

We require information from authors about some types of materials, experimental systems and methods used in many studies. Here, indicate whether each material, system or method listed is relevant to your study. If you are not sure if a list item applies to your research, read the appropriate section before selecting a response.

Materials & experimental systems

n/a	Involved in the study	
	x	Antibodies
	x	Eukaryotic cell lines
x		Palaeontology and archaeology
x		Animals and other organisms
x		Clinical data
x		Dual use research of concern
x		Plants

Methods

n/a	Involved in the study	
x		ChIP-seq
x		Flow cytometry
x		MRI-based neuroimaging

Antibodies

Antibodies used

The detailed list of antibodies including their source,catalog numbers and dilutions are mentioned here as follows as well as in the Supplementary table as well:
AF9 Polyclonal antibody(Bethyl Laboratories,A300-596A) (Dilution 1 : 2000)
ATM antibody (Cell Signaling Technology,clone D2E2, 2873) (Dilution 1 : 2000)
β-Actin antibody(BioBharati Life Science,BB-AB0024) (Dilution 1 : 5000)
Acetylated-Lysine antibody(Cell Signaling Technology,9441) (Dilution 1 : 2000)
BMI1 antibody(Cell Signaling Technology,D20B7,6964) (Dilution 1 : 1500)
CDK9 antibody(Santa Cruz Biotechnology,sc-13130) (Dilution 1 : 2000)
CCNT1 antibody(Santa Cruz Biotechnology,sc-271348) (Dilution 1 : 2000)
DNA-PKc antibody(Cell Signaling Technology,E6U3A,38168) (Dilution 1 : 2000)
ELL antibody(Cell Signaling Technology,D7N6U,14468) (Dilution 1 : 2000)
ENL antibody(Cell Signaling Technology,DNM4B,14893) (Dilution 1 : 2000)
FLAG epitope (Sigma, F7425) (Dilution 1 : 5000)
GFP antibody(BioBharati Life Science,BB-AB0065) (Dilution 1 : 2000)
GST antibody(Santa Cruz Biotechnology,sc-138) (Dilution 1 : 2000)
HA epitope (Santa Cruz Biotechnology,sc-57592) (Dilution 1 : 200)
His epitope (SantaCruz Biotechnology,sc-8036) (Dilution 1 : 1000)
HDAC5 antibody(Cell Signaling Technology ,E6G3N,98329) (Dilution 1 : 2000)
Histone H3 antibody(Cell Signaling Technology,D1H2,4499) (Dilution 1 : 5000)
Histone H2A.X antibody(Cell Signaling Technology ,D17A3,#7631) (Dilution 1 : 5000)
Phospho-Histone H2A.X antibody (Ser139) (20E3)(Cell Signaling Technology,9718) (Dilution 1 : 5000)
Ku70 antibody(Cell Signaling Technology,D10A7,4588) (Dilution 1 : 2000)
Ku80 antibody(Cell Signaling Technology,2753) (Dilution 1 : 2000)
Myc antibody (Santa Cruz Biotechnology,sc-40) (Dilution 1 : 1000)
PCAF antibody(Santa Cruz Biotechnology,sc-13124) (Dilution 1 : 2000)
TBP antibody(Bethyl Laboratories,A301-229A) (Dilution 1 : 2000)
TAF1 antibody(Cell Signaling Technology,D6J8B,12781) (Dilution 1 : 1000)
TAF5 antibody(Bethyl Laboratories,A303-685A) (Dilution 1 : 1000)
TAF6 antibody(Bethyl Laboratories,A301-275A) (Dilution 1 : 2000)
TAF7 antibody(Santa Cruz Biotechnology,sc-101167) (Dilution 1 : 2000)
S/T-Q Phospho antibody (Cell Signaling Technology,9607) (Dilution 1 : 2000)
AF9-K339Ac antibody(BioBharati Life Science,BB-SAP50) (Dilution 1 : 2000)
Phospho-Rpb1 CTD (Ser2) antibody (Cell Signaling Technology,E1Z3G,13499) (Dilution 1 : 2000)
Phospho-Rpb1 CTD (Ser5) antibody(Cell Signaling Technology,D9N5I, 13523) (Dilution 1 : 2000)
Normal Rabbit IgG antibody(Cell Signaling Technology,2729) (Dilution 1 : 2000)
Normal Mouse IgG antibody(Cell Signaling Technology,5415) (Dilution 1 : 1000)

Validation

All these antibodies, except AF9 K339Ac-specific antibody(BioBharati Life Science,BB-SAP50) were commercially obtained and validated by vendors and multiple published studies by us and others, see manufacture's website for references. The validation details of AF9(K339Ac-specific) antibody are mentioned within the manuscript. We are also listing the Research Resource Identifiers (RRIDs) for each antibody here for authentication.
AF9 Rabbit Polyclonal antibody (Bethyl Laboratories, A300-596A) RRID: AB_495519
ATM Rabbit Monoclonal antibody (Cell Signaling Technology, 2873) RRID: AB_2062659
β-Actin Rabbit Polyclonal antibody (BioBharati Life Science,BB-AB0024) was validated by western blotting ((https://biobharti.com/anti- β-actin -antibody/)
Acetylated-Lysine antibody (Cell Signaling Technology,9441) RRID:AB_331805
BMI1 Rabbit Monoclonal antibody (Cell Signaling Technology,6964) RRID:AB_10828713
CDK9 Mouse Monoclonal antibody (Santa Cruz Biotechnology,sc-13130) RRID:AB_627245
CCNT1 Mouse Monoclonal antibody (Santa Cruz Biotechnology,sc-271348) RRID:AB_10608086
DNA-PKc Rabbit Monoclonal antibody (Cell Signaling Technology,E6U3A,38168) RRID:AB_2799128
ELL Rabbit Monoclonal antibody (Cell Signaling Technology,D7N6U,14468) RRID:AB_2798489
ENL Rabbit Monoclonal antibody (Cell Signaling Technology,DNM4B,14893) RRID:AB_2798636

FLAG Rabbit Polyclonal antibody(Sigma, F7425) RRID:AB_439687
GFP Rabbit Polyclonal antibody(BioBharati Life Science,BB-AB0065) was validated by western blotting(https://biobharti.com/anti-gfp-antibody/)
GST Mouse Monoclonal antibody(Santa Cruz Biotechnology,sc-138) RRID:AB_627677
HA-Tag Mouse Monoclonal antibody(Santa Cruz Biotechnology,sc-57592) RRID:AB_629568
His-Tag Mouse Monoclonal antibody(SantaCruz Biotechnology,sc-8036) RRID:AB_627727
HDAC5 Mouse Monoclonal antibody(Cell Signaling Technology ,E6G3N,98329) RRID:AB_10559747
Histone H3 Rabbit Monoclonal antibody(Cell Signaling Technology,D1H2,4499) RRID:AB_10544537
Histone H2A.X Rabbit Monoclonal antibody(Cell Signaling Technology ,D17A3,7631) RRID:AB_10860771
Phospho-Histone H2A.X Rabbit Monoclonal antibody (Ser139) (20E3)(Cell Signaling Technology,9718) RRID:AB_2118009
Ku70 Rabbit Monoclonal antibody(Cell Signaling Technology,D10A7,4588) RRID:AB_11179211
Ku80 Rabbit Monoclonal antibody(Cell Signaling Technology,2753) RRID:AB_2257526
Myc Mouse Monoclonal antibody (Santa Cruz Biotechnology,sc-40) RRID:AB_627268
PCAF Mouse Monoclonal antibody(Santa Cruz Biotechnology,sc-13124) RRID:AB_2128417
TBP Rabbit Monoclonal antibody(Bethyl Laboratories,A301-229A) RRID:AB_890661
TAF1 Rabbit Monoclonal antibody(Cell Signaling Technology,D6J8B,12781) RRID:AB_2798025
TAF5 Rabbit Monoclonal antibody(Bethyl Laboratories,A303-685A) RRID:AB_11204926
TAF6 Rabbit Monoclonal antibody(Bethyl Laboratories,A301-275A) RRID:AB_938019
TAF7 Mouse Monoclonal antibody(Santa Cruz Biotechnology,sc-101167) RRID:AB_2199332
S/T-Q Phospho Rabbit Monoclonal antibody (Cell Signaling Technology,9607) RRID:AB_10889739
Phospho-Rpb1 CTD (Ser2) Rabbit Monoclonal antibody (Cell Signaling Technology,E1Z3G,13499) RRID:AB_2798238
Phospho-Rpb1 CTD (Ser5) Rabbit Monoclonal antibody(Cell Signaling Technology,D9N5I, 13523) RRID:AB_2798246
Normal Rabbit IgG antibody(Cell Signaling Technology,2729) RRID:AB_1031062
Normal Mouse IgG antibody(Cell Signaling Technology,5415) RRID:AB_10829607

Eukaryotic cell lines

Policy information about cell lines and Sex and Gender in Research

Cell line source(s)	HEK293T and HeLa cell lines were used in this study. Both these cell lines were obtained from ATCC.
Authentication	Certificate of analysis for HEK293T and HeLa cells were provided by the vendors. STR profiling was done.
Mycoplasma contamination	Both these cell lines were periodically checked for mycoplasma contamination using MycoStrip™-Mycoplasma Detection Kit . Further, cells were grown in media containing small amount of mycoplasma contamination preventing agents. All the cell lines used for our experiments tested negative for mycoplasma contamination.
Commonly misidentified lines (See ICLAC register)	No commonly misidentified lines were observed.

Palaeontology and Archaeology

Specimen provenance	*Provide provenance information for specimens and describe permits that were obtained for the work (including the name of the issuing authority, the date of issue, and any identifying information). Permits should encompass collection and, where applicable, export.*
Specimen deposition	*Indicate where the specimens have been deposited to permit free access by other researchers.*
Dating methods	*If new dates are provided, describe how they were obtained (e.g. collection, storage, sample pretreatment and measurement), where they were obtained (i.e. lab name), the calibration program and the protocol for quality assurance OR state that no new dates are provided.*

☐ Tick this box to confirm that the raw and calibrated dates are available in the paper or in Supplementary Information.

Ethics oversight	*Identify the organization(s) that approved or provided guidance on the study protocol, OR state that no ethical approval or guidance was required and explain why not.*

Note that full information on the approval of the study protocol must also be provided in the manuscript.

Animals and other research organisms

Policy information about studies involving animals; ARRIVE guidelines recommended for reporting animal research, and Sex and Gender in Research

Laboratory animals	*For laboratory animals, report species, strain and age OR state that the study did not involve laboratory animals.*
Wild animals	*Provide details on animals observed in or captured in the field; report species and age where possible. Describe how animals were caught and transported and what happened to captive animals after the study (if killed, explain why and describe method; if released, say where and when) OR state that the study did not involve wild animals.*
Reporting on sex	*Indicate if findings apply to only one sex; describe whether sex was considered in study design, methods used for assigning sex. Provide data disaggregated for sex where this information has been collected in the source data as appropriate; provide overall numbers in this Reporting Summary. Please state if this information has not been collected. Report sex-based analyses where performed, justify reasons for lack of sex-based analysis.*
Field-collected samples	*For laboratory work with field-collected samples, describe all relevant parameters such as housing, maintenance, temperature, photoperiod and end-of-experiment protocol OR state that the study did not involve samples collected from the field.*
Ethics oversight	*Identify the organization(s) that approved or provided guidance on the study protocol, OR state that no ethical approval or guidance was required and explain why not.*

Note that full information on the approval of the study protocol must also be provided in the manuscript.

Clinical data

Policy information about clinical studies

All manuscripts should comply with the ICMJEguidelines for publication of clinical research and a completedCONSORT checklist must be included with all submissions.

Clinical trial registration	*Provide the trial registration number from ClinicalTrials.gov or an equivalent agency.*
Study protocol	*Note where the full trial protocol can be accessed OR if not available, explain why.*
Data collection	*Describe the settings and locales of data collection, noting the time periods of recruitment and data collection.*
Outcomes	*Describe how you pre-defined primary and secondary outcome measures and how you assessed these measures.*

Dual use research of concern

Policy information about dual use research of concern

Hazards

Could the accidental, deliberate or reckless misuse of agents or technologies generated in the work, or the application of information presented in the manuscript, pose a threat to:

No	Yes	
☐	☐	Public health
☐	☐	National security
☐	☐	Crops and/or livestock
☐	☐	Ecosystems
☐	☐	Any other significant area

Experiments of concern

Does the work involve any of these experiments of concern:

No	Yes	
☐	☐	Demonstrate how to render a vaccine ineffective
☐	☐	Confer resistance to therapeutically useful antibiotics or antiviral agents
☐	☐	Enhance the virulence of a pathogen or render a nonpathogen virulent
☐	☐	Increase transmissibility of a pathogen
☐	☐	Alter the host range of a pathogen
☐	☐	Enable evasion of diagnostic/detection modalities
☐	☐	Enable the weaponization of a biological agent or toxin
☐	☐	Any other potentially harmful combination of experiments and agents

Plants

Seed stocks	*Report on the source of all seed stocks or other plant material used. If applicable, state the seed stock centre and catalogue number. If plant specimens were collected from the field, describe the collection location, date and sampling procedures.*
Novel plant genotypes	*Describe the methods by which all novel plant genotypes were produced. This includes those generated by transgenic approaches, gene editing, chemical/radiation-based mutagenesis and hybridization. For transgenic lines, describe the transformation method, the number of independent lines analyzed and the generation upon which experiments were performed. For gene-edited lines, describe the editor used, the endogenous sequence targeted for editing, the targeting guide RNA sequence (if applicable) and how the editor was applied.*
Authentication	*Describe any authentication procedures for each seed stock used or novel genotype generated. Describe any experiments used to assess the effect of a mutation and, where applicable, how potential secondary effects (e.g. second site T-DNA insertions, mosiacism, off-target gene editing) were examined.*

ChIP-seq

Data deposition

☐ Confirm that both raw and final processed data have been deposited in a public database such as GEO.

☐ Confirm that you have deposited or provided access to graph files (e.g. BED files) for the called peaks.

Data access links<br>*May remain private before publication.*	*For "Initial submission" or "Revised version" documents, provide reviewer access links. For your "Final submission" document, provide a link to the deposited data.*
Files in database submission	*Provide a list of all files available in the database submission.*
Genome browser session<br>(e.g. UCSC)	*Provide a link to an anonymized genome browser session for "Initial submission" and "Revised version" documents only, to enable peer review. Write "no longer applicable" for "Final submission" documents.*

Methodology

Replicates	*Describe the experimental replicates, specifying number, type and replicate agreement.*
Sequencing depth	*Describe the sequencing depth for each experiment, providing the total number of reads, uniquely mapped reads, length of reads and whether they were paired- or single-end.*
Antibodies	*Describe the antibodies used for the ChIP-seq experiments; as applicable, provide supplier name, catalog number, clone name, and lot number.*
Peak calling parameters	*Specify the command line program and parameters used for read mapping and peak calling, including the ChIP, control and index files used.*
Data quality	*Describe the methods used to ensure data quality in full detail, including how many peaks are at FDR 5% and above 5-fold enrichment.*
Software	*Describe the software used to collect and analyze the ChIP-seq data. For custom code that has been deposited into a community repository, provide accession details.*

Flow Cytometry

Plots

Confirm that:

- [ ] The axis labels state the marker and fluorochrome used (e.g. CD4-FITC).
- [ ] The axis scales are clearly visible. Include numbers along axes only for bottom left plot of group (a 'group' is an analysis of identical markers).
- [ ] All plots are contour plots with outliers or pseudocolor plots.
- [ ] A numerical value for number of cells or percentage (with statistics) is provided.

Methodology

Sample preparation	*Describe the sample preparation, detailing the biological source of the cells and any tissue processing steps used.*
Instrument	*Identify the instrument used for data collection, specifying make and model number.*
Software	*Describe the software used to collect and analyze the flow cytometry data. For custom code that has been deposited into a community repository, provide accession details.*
Cell population abundance	*Describe the abundance of the relevant cell populations within post-sort fractions, providing details on the purity of the samples and how it was determined.*
Gating strategy	*Describe the gating strategy used for all relevant experiments, specifying the preliminary FSC/SSC gates of the starting cell population, indicating where boundaries between "positive" and "negative" staining cell populations are defined.*

- [ ] Tick this box to confirm that a figure exemplifying the gating strategy is provided in the Supplementary Information.

Magnetic resonance imaging

Experimental design

Design type	*Indicate task or resting state; event-related or block design.*
Design specifications	*Specify the number of blocks, trials or experimental units per session and/or subject, and specify the length of each trial or block (if trials are blocked) and interval between trials.*
Behavioral performance measures	*State number and/or type of variables recorded (e.g. correct button press, response time) and what statistics were used to establish that the subjects were performing the task as expected (e.g. mean, range, and/or standard deviation across subjects).*

Acquisition

Imaging type(s)	*Specify: functional, structural, diffusion, perfusion.*
Field strength	*Specify in Tesla*
Sequence & imaging parameters	*Specify the pulse sequence type (gradient echo, spin echo, etc.), imaging type (EPI, spiral, etc.), field of view, matrix size, slice thickness, orientation and TE/TR/flip angle.*
Area of acquisition	*State whether a whole brain scan was used OR define the area of acquisition, describing how the region was determined.*

Diffusion MRI ☐ Used ☐ Not used

Preprocessing

Preprocessing software	*Provide detail on software version and revision number and on specific parameters (model/functions, brain extraction, segmentation, smoothing kernel size, etc.).*
Normalization	*If data were normalized/standardized, describe the approach(es): specify linear or non-linear and define image types used for transformation OR indicate that data were not normalized and explain rationale for lack of normalization.*
Normalization template	*Describe the template used for normalization/transformation, specifying subject space or group standardized space (e.g. original Talairach, MNI305, ICBM152) OR indicate that the data were not normalized.*
Noise and artifact removal	*Describe your procedure(s) for artifact and structured noise removal, specifying motion parameters, tissue signals and physiological signals (heart rate, respiration).*

Volume censoring | *Define your software and/or method and criteria for volume censoring, and state the extent of such censoring.*

Statistical modeling & inference

Model type and settings | *Specify type (mass univariate, multivariate, RSA, predictive, etc.) and describe essential details of the model at the first and second levels (e.g. fixed, random or mixed effects; drift or auto-correlation).*

Effect(s) tested | *Define precise effect in terms of the task or stimulus conditions instead of psychological concepts and indicate whether ANOVA or factorial designs were used.*

Specify type of analysis: ☐ Whole brain ☐ ROI-based ☐ Both

Statistic type for inference | *Specify voxel-wise or cluster-wise and report all relevant parameters for cluster-wise methods.*

(See Eklund et al. 2016)

Correction | *Describe the type of correction and how it is obtained for multiple comparisons (e.g. FWE, FDR, permutation or Monte Carlo).*

Models & analysis

n/a	Involved in the study
☐	☐ Functional and/or effective connectivity
☐	☐ Graph analysis
☐	☐ Multivariate modeling or predictive analysis

Functional and/or effective connectivity | *Report the measures of dependence used and the model details (e.g. Pearson correlation, partial correlation, mutual information).*

Graph analysis | *Report the dependent variable and connectivity measure, specifying weighted graph or binarized graph, subject- or group-level, and the global and/or node summaries used (e.g. clustering coefficient, efficiency, etc.).*

Multivariate modeling and predictive analysis | *Specify independent variables, features extraction and dimension reduction, model, training and evaluation metrics.*
